# A Novel 3-Dimensional Printed Nanoceramic Hybrid Resin Fixed Lingual Retainer: Characterization and Mechanical Tests

**DOI:** 10.1155/2024/3540846

**Published:** 2024-10-15

**Authors:** Noor Salam Alnuaimy, Akram Faisal Alhuwaizi

**Affiliations:** Department of Orthodontics, College of Dentistry, University of Baghdad, Baghdad, Iraq

**Keywords:** 3D-printed retainers, fixed retainer, lingual retainer, orthodontic retention

## Abstract

**Introduction:** An innovative retention protocol was developed to create a new 3D-printed fixed retainer employing SprintRay OnX nanoceramic hybrid resin. The feasibility and usability of the retainer were subsequently evaluated.

**Methods:** Identification and characterization of SprintRay OnX was done using Fourier transform infrared spectroscopy (FTIR), scanning electron microscopy with energy dispersive X-ray (SEM-EDX), field emission scanning electron microscope (FE-SEM), X-ray diffraction (XRD), and flexural strength. Load–deflection and pull-out tests were conducted on the 3D-printed straight wires, with three distinct cross-sectional geometries: round (1 mm), oval (1 mm × 1.5 mm) and semielliptical (1 mm × 1.5 mm). Twisted G&H and coaxial Respond stainless steel multistrand retainers were used for comparison. In the load–deflection test, a three-point bending test (3PBT) was employed. For the pull-out test, the retainer wire was inserted into the composite, which was placed in a centrally located hole of an acrylic block; the retainer wire was subjected to a tensile force along its long axis.

**Results:** Characteristic bands close to those of PMMA were observed in the FTIR spectra. SEM-EDX and XRD revealed a crystalline material with homogeneously distributed Yb element signals (19.4%). On FE-SEM micrographs, small clumps were displayed on smooth surfaces. The flexural strength and the flexural modulus were, respectively, 142.48 MPa and 7.842 GPa. All groups of 3D-printed wires exhibited significantly higher load–deflection levels than the multistrand wires (MSWs). Concerning pull-out forces, they fell in between twisted G&H (96 N) and coaxial Respond (48.09 N) retainer wires. The 3D-printed wires fractured cohesively without detachment from the adhesive, suggesting that the chemical bond was adequate for satisfactory wire integration, yet the wire's strength was compromised. Concerning the cross-sectional geometry, the load–deflection and the pull-out forces of 3D-printed oval and semielliptical wires were significantly higher than that of 3D-printed round wires, which was attributed to the larger cross-sections of the wires.

**Conclusion:** Oval and semielliptical 3D-printed wires offered favorable features as lingual retainers.

## 1. Introduction

Over a century ago, Norman Kingsley asserted that “the success of orthodontia as a science and art as now lies in retainer” [1]. Functional and esthetic occlusion should not imply the end of the orthodontic treatment [2]. Posttreatment changes may occur due to relapse, that is, the propensity of teeth to revert to their pretreatment position or normal age-related changes [3, 4]. Retention, the maintenance of teeth in their positions following orthodontic treatment, is an integral phase of orthodontic practice and one of the most challenging aspects [5]. The necessity of indefinite retention is now widely accepted [6]. Recent systematic reviews revealed that fixed retainers were more effective than removable alternatives over long-term follow-up [7–10]. A shift from removable to fixed retention became prevalent in clinical practice throughout the past century [11].

As a fixed orthodontic retainer, stainless steel multistrand wires (MSWs) bonded to the anterior teeth continue to be the gold standard [10, 12]. The wire flexibility facilitates physiological tooth mobility and reduces bonding composite stress while the teeth are splinted [13]. Nevertheless, the failure rates ranged from 7.3% to 50% and were more frequent in the maxilla than in the mandible [14]. Debonding or retainer fracture might cause this common complication [4]. On the other hand, bonded retainers lead to more significant dental plaque and calculus accumulation in the vicinity of retainers that may not cause serious detrimental periodontal effects [15, 16]. Inadvertent tooth movement—unexplained, aberrant, unexpected, unwanted, or undesirable—can be a more serious sequel. It can occur with the intact retainer *in situ* without detachment or fracture [15, 17]. Wire distortion induced by insufficient passivity of the bonded wire or activation during the retention due to occlusal contact, habits, hard food particles, or flossing may generate forces capable of moving teeth [4, 17]. Stainless steel, used in most bonded retainers, may be hazardous to general health; MRI necessitates metal removal to avoid overheating and image artifacts, and it is contraindicated for patients with metal allergies [18, 19].

Recent CAD/CAM subtractive manufacturing trials for fixed retainers, including zirconium [20, 21], PEEK [21–26], and NiTi retainers [12, 21, 27–32], have overcome some of these concerns. However, additive manufacturing technology builds the desired object from raw material layer by layer in a specified pattern, enabling more precise and complex objects. 3D-printing outperforms subtractive technologies in terms of cost-effectiveness, reduced wasted raw material, manufacturing speed, high-quality production and precision, and complex and sophisticated shapes and geometries, which other methods cannot make [33, 34]. On the other hand, methacrylate-based resin, frequently utilized in dental applications, does not have favorable physicomechanical properties. Consequently, the prosthesis may be susceptible to fracture by high masticatory forces. Hence, the research area has shifted its focus to the reinforcement of nanoparticles such as ceramic or metal oxides synthesizing various nanocomposites to enhance mechanical properties [35–37]. This study developed a novel 3D-printed fixed retainer using SprintRay OnX nanoceramic hybrid resin (SprintRay, Los Angeles, California, USA) to address most drawbacks and mitigate the risks associated with conventional retainers. Being entirely metal-free, it can be used for patients with metal allergies or MRI. Intraoral scanning and digital design provide a high degree of customization, which can impart optimal placement and interproximal adjustment, no need for wire measurement or bending, operator skill, and consequently, reduced appointment durations. Digital archiving and retrieval make remanufacturing a broken or detached retainer as simple as duplicating the retainer. These assumed advantages over MSW retainers may enhance permanent retention in modern orthodontics. The material characteristics, fabrication process, and *in vitro* mechanical testing of the 3D-printed wires were evaluated to determine their suitability as contemporary lingual fixed retainers.

## 2. Methods

### 2.1. Specimen Preparation

The specimens' designs were built with Microsoft 3D Builder and saved as STL files (the standard triangulated language). They were then remeshed using the Autodesk Meshmixer program to enhance STL triangulation and printing quality. The 3D-printing was performed using the DLP-based printer (Pro 95 Printer, SprintRay, Los Angeles, California, USA), where the OnX printing specs were accessible. All printing and postprocessing steps were implemented following the manufacturer's instructions. The samples' STL files were loaded into RayWare Automated Dental 3D Printing software (Version 2.9.1). The specimens were printed vertically from the build platform [38, 39]. The printing supports were automatically created with minimal dimensions to produce weak areas requiring little reduction. The resin bottle (shade A1) was mixed thoroughly before each print using an LC-3D Mixer. Each specimen was built layer by layer with 50 microns of thickness. All print jobs in the study were executed using a single resin package (Lot S22C21XA1) at room temperature (25 ± 2°C).

The printed samples were kept on the build platform for 5–10 min to allow the extra resin to drip off into the tank; then, they were removed using the print-removal tool. Subsequently, a light spray of 91% high-purity isopropyl alcohol was used to clean and wash the samples. Compressed air was then blasted to dislodge as much uncured resin residue as possible. The cleaning cycle was repeated seven times. After drying, the samples were adequately postcured using the ProCure postcuring system (SprintRay, Los Angeles, California, USA) to ensure complete polymerization and maximize their strength and accuracy. A 90-W LED array with a broad-spectrum wavelength range of 365–405 nm and light intensity of 28.8 mW/cm^2^ was employed to postcure the sample in a heated environment at 60°C for 60 min. After that, the supports were cut as close to the item as possible using a round diamond disc.

**Figure 1:**
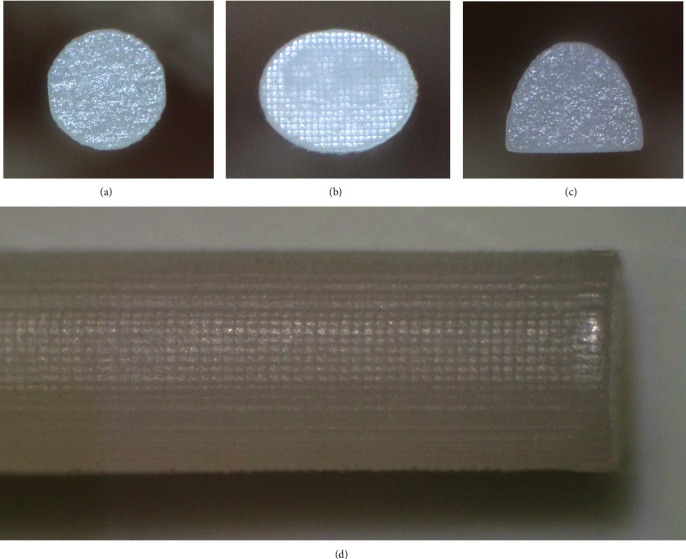
Straight 3D-printed wires under stereomicroscope (×10 magnification): (A) round, (B) oval, and (C) semielliptical cross-sectional geometries, and (D) the surface texture of a straight 3D-printed round wire.

The specimens were inspected visually for manufacturing flaws. The wire's surface was examined under a stereomicroscope (×10 magnification) to assess its quality and exclude the presence of any imperfection or air bubbles (Figure 1). The dimensions of each sample were measured using a digital caliper (0.01 mm accuracy) to ensure sufficient printing accuracy and precision. Any defective samples were excluded. To eliminate contaminants, specimens were cleaned in an ultrasonic chamber using ethanol followed by distilled water for 5 min.

### 2.2. Material Identification and Characterization

The 3D-printed resin (Table 1) was characterized using various methods: Fourier transform infrared spectroscopy (FTIR), scanning electron microscopy with energy dispersive X-ray (SEM-EDX), field emission scanning electron microscope (FE-SEM), X-ray diffraction (XRD), and flexural strength.

The FTIR was conducted using an ALPHA-P spectrometer (Burker, Billerica, MA, USA) with an attenuated total reflection (ATR probe). The FTIR-ATR diamond crystal was employed to determine the material's composition and crystal chemistry by identifying the distinctive functional groups. Three disc-shaped specimens (10 mm × 2 mm) were analyzed. The specimen was pressed against the lens for maximum infrared signal intensity. Spectra were acquired in transmittance mode within the 400–4000 cm^−1^ range, with a resolution of 4 cm^−1^ and an accuracy of >0.05 cm^−1^ using the Opus 8.7 SP2 spectroscopy software.

The SEM-EDX analysis was used to investigate the surface morphology, identify the chemical elemental composition of the material, and quantify the elemental distribution. Three disc-shaped specimens (10 mm × 2 mm) were examined. The specimen was placed on a carbon disc using a double-sided adhesive tape. The SEM pictures were acquired with backscattered electron emission at 30 kV accelerating voltage using Axia ChemiSEM (Thermo Fisher Scientific, USA).

The nanostructure of the specimens was characterized by analyzing their surface morphologies in the acquired FE-SEM images. Using a YKY plasma sputtering coater, three disc-shaped specimens (10 mm × 2 mm) were coated with a 15 nm-thick gold–palladium alloy for conductivity and imaging resolution. The specimen was affixed to SEM stub-holders using double-sided adhesive carbon tape and viewed in the InspectTM F50 FE-SEM (FEI, USA) at an accelerating voltage of 30 kV and a working distance of 14.4 mm.

The XRD-6000 diffractometer system (Lab X, Shimadzu, Japan) was utilized to analyze the crystallinity and structural characteristics of the material using three cuboid-shaped specimens (30 mm × 30 mm × 1 mm). The system was equipped with Cu K*α* radiation with a continuous scan mode at a 2*θ* range between 10° and 70° and operated at 40 kV and 40 mA at a scanning speed of about 5°/min.

**Figure 2:**
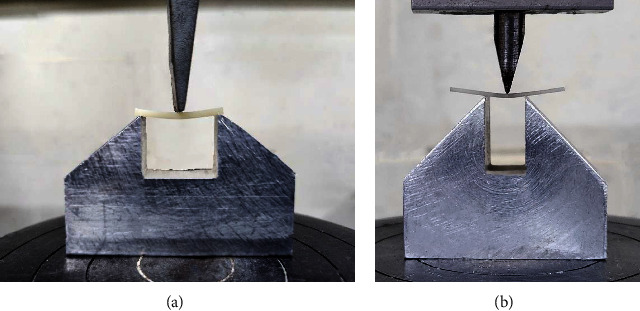
Flexural strength test (A) load–deflection test (B) using Instron machine.

For flexural properties, the three-point bending test (3PBT) protocol was derived from the technical standard ISO.10477 [40]. Before conducting the test, 10 rectangular beams (2 mm × 2 mm × 25 mm) were held in water in an incubator at 37°C for 24 h [41]. A chisel end metal plunger with a 3 mm diameter and a U-shaped metal fixture were made. Tinius Olsen universal testing machine (H50KT, England) was equipped with a 10 kN load cell [42], and the metal plunger was installed on top. Each specimen was mounted on the fixture, with a 20 mm distance between two round parallel supports. Continuously increasing uniaxial vertical load was applied to the specimen's center with a crosshead speed of 1 mm/min till fracture. The maximal force before fracture was recorded in Newtons (N; Figure 2A). Flexural strength (*σ*) in MPa and flexural modulus (*E*) in GPa were determined using the subsequent equations, respectively:

  $$\begin{array}{c} \sigma =3Fl/2wh^{2} \end{array}$$

  $$\begin{array}{c} E=Fl^{3}/4wh^{3}d \end{array}$$

where *F* represents the maximum force to fracture in N; *l*, the distance between the supports in mm; *w*, the width of the specimen in mm; *h*, the height of the specimen in mm; and *d*, the deflection of the specimen in mm at load *F*.

### 2.3. Mechanical Tests of the 3D-Printed Straight Wires

Retainer wires with three distinct cross-sectional geometries (round, oval, and semielliptical) were printed. Straight pieces of the control wires were cut to the same lengths. Five groups of 10 straight lingual retainer wires were prepared as follows (the same groups for load–deflection and pull-out tests):

**Group I (3D Round):** 3D-printed round lingual retainer wires (1 mm diameter).

**Group II (3D Oval):** 3D-printed oval lingual retainer wires (1 mm thickness and  ^*∗*^1.5 mm width).

**Group III (3D Semielliptical):** 3D-printed semielliptical lingual retainer wires (1 thickness and  ^*∗*^1.5 mm width).

**Group IV (Twisted G&H):** 0.0195″ round seven-strand twisted stainless steel retainer wires (G&H multistrand retainer wire, G&H Orthodontics, Franklin, IN, USA).

**Group V (Coaxial Respond):** 0.0195″ round six-strand coaxial stainless steel dead-soft retainer wires (Respond Archwire, Ormco Corp., Orange, CA, USA).

#### 2.3.1. Load–Deflection Test

The load–deflection test was done following ISO.15841 [43] regulation. 3PBT was conducted using a Tinius Olsen universal testing machine under dry circumstances and at room temperature. A chisel end metal plunger (1 mm radius of curvature at its free end) and a U-shape block metal fixture were constructed [44, 45]. Each sample (30 mm in length) was positioned on the fixture's supports to be centered on the inter-support distance (10 mm). The striker (plunger) was attached to the top moveable head of the Instron machine and positioned such that the tip touched the midspan of the wire without deflection. Before each test, the load cell was adapted and calibrated. Each sample was exposed to a vertical force with a 10 N load cell [46] at a crosshead speed of 1 mm/min until deflected by 1.5 mm. Forces for the deflection were recorded directly onto the computer connected to the equipment. A load–deflection curve depicting wire deflection on the *x*-axis and force exerted on the *y*-axis was drawn for each sample. Readings were taken at 0.1, 0.5, 1, and 1.5 mm [19, 47–49] (Figure 2B).

#### 2.3.2. Pull-Out Test

**Figure 3:**
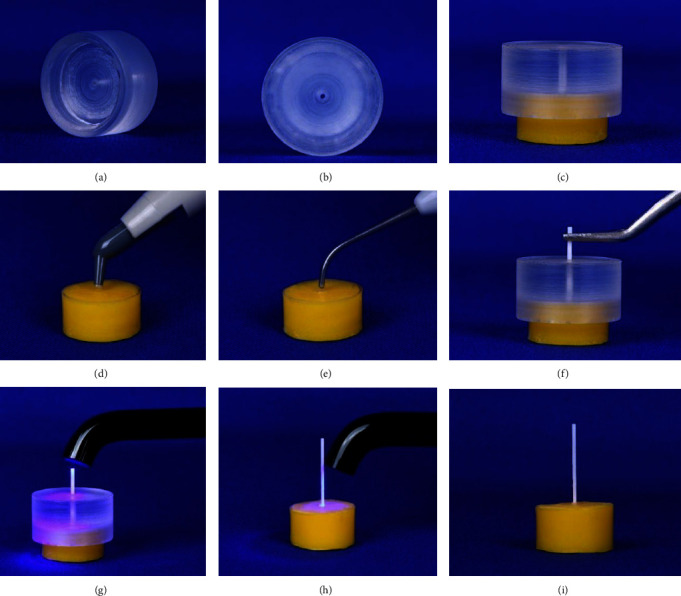
Procedure of retainer wire bonding to the pull-out block: (A–C) clear guiding block, (D) adding adhesive to the hole, (E) condensing the adhesive, (F) positioning the guide and the wire, (G) initial tacking of the adhesive, (H) curing the adhesive, and (I) the block with the retainer wire bonded.

The study's design and set-up were adopted from the research conducted by Bearn et al. [50]. Fifty cylindrical acrylic blocks (25 mm diameter and 10 mm height) were made [50, 51] and assigned to five test groups. A 2 mm width by 3 mm depth hole was drilled in the center of the upper surface of each acrylic block [22, 50–52] using a large round carbide bur adapted to the portable engine. It was oriented perpendicularly using the vertical arm of the dental surveyor. Water cooling spray was used to protect against thermal damage during drilling, and then acrylic debris was cleaned. The drilled hole's dimensions approximated the composite size used clinically to bond the wire to the tooth in a bonded retainer and the length of the embedded wire in the composite [50, 51]. Transbond XT adhesive primer was applied to the walls and floor of the hole. The hole was then filled with Transbond LR composite (3M Unitek, Monrovia, California, USA), a highly filled light cure adhesive composite designed for bonding lingual retainers. A second clear guiding block, with slightly bigger dimensions than the test block, with a central hole, was specially designed and constructed [50, 51]. The acrylic guide was fitted over the prepared block to guarantee perfect wire placement [50, 51]. The retainer wire (20 mm length) passed through the central hole, so it was parallel to the block's long axis, and one end was embedded in composite at the hole's center. Initial tacking was done while the guide was in place, and then the adhesive was cured for 10 s with Elipar™ DeepCure-L light emitting diode (LED) curing unit (3M ESPE, USA) with 430–480 nm wavelength and 1470 mW/cm^2^ light intensity (Figure 3). The test samples were kept in distilled water and stored in the incubator at 37°C for around 24 h before running the test [22, 50].

**Figure 4:**
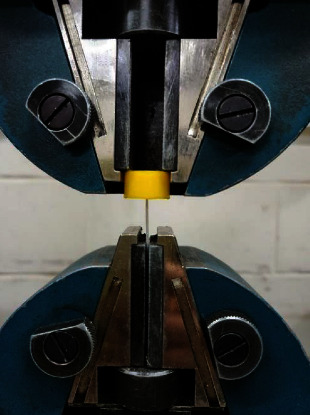
The test block was attached to the movable upper jaw, while the wire was firmly grabbed by the lower fixed jaw of the Instron machine during the pull-out test.

The block was attached to the upper movable jaw of the Tinius Olsen universal testing machine with a 50 kN load cell, while the lower fixed jaw firmly grabbed the wire. The pull-out (tensile) force was vertically applied along the long axis of the sample at 10 mm/min speed. The maximum force measured in N is referred to as the ultimate force of failure. The test persisted until the wire broke or detached from the composite [22, 51, 52] (Figure 4).

Statistical analyses were conducted utilizing SPSS 26.0 (SPSS Inc., Chicago, IL) for load–deflection and pull-out tests at a significance level of *p*  < 0.05. The data were analyzed using the Shapiro–Wilks normality test and Levene's variance homogeneity test. Since the data were normally distributed with unequal variances, the Welch *F* test, alongside the ANOVA and Games–Howell post hoc multicomparison test, were performed.

## 3. Results

### 3.1. Material Identification and Characterization

**Figure 5:**
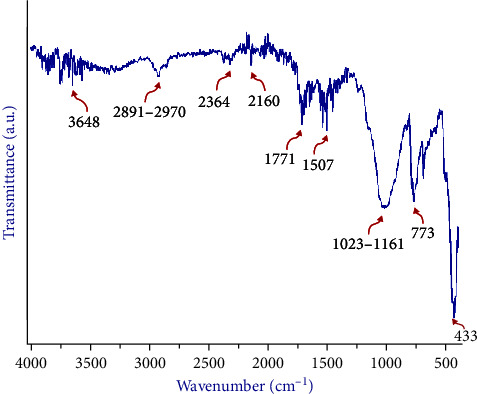
FTIR spectrum analysis of atomic peaks and functional groups of 3D-printed OnX material.

The FTIR spectra (Figure 5) demonstrated the broadest and strongest bands at 1023–1161 cm^−1^ and 433 cm^−1^. The infrared reflectance spectrum revealed several distinct bands close to the main characteristic peaks and made up the fingerprint of the PMMA spectrum, indicating the shared properties of methacrylic acid. The corresponding depicted spectra peeks were as follows: 1023 cm^−1^ bands verified C–O stretching vibrations; the 1161 cm^−1^ band could be ascribed to the C═O bending mode, representing carbonyl groups; 773 cm^−1^ indicated stretching vibrations of the C–H bond; 1771 cm^−1^ indicated stretching vibrations of the ester carbonyl groups; and 2970–2891 cm^−1^ could be attributed to the stretching C–H vibration, both symmetric and asymmetric. The high-intensity peaks at 433 cm^−1^ and 1507 cm^−1^, representing C═C stretching mode, had shifted slightly from pure PMMA, suggesting a possible interaction with the added nanoparticles. The results of the spectrum analyses for the three specimens were similar.

**Figure 6:**
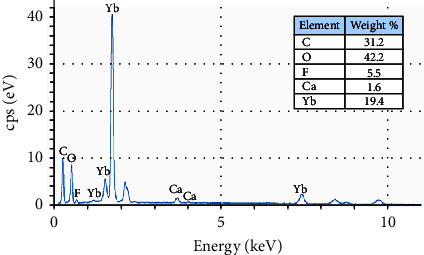
EDX plot displaying the elemental analysis of the 3D-printed OnX material and a summary of weight-wise percentages of the material elements.

**Figure 7:**
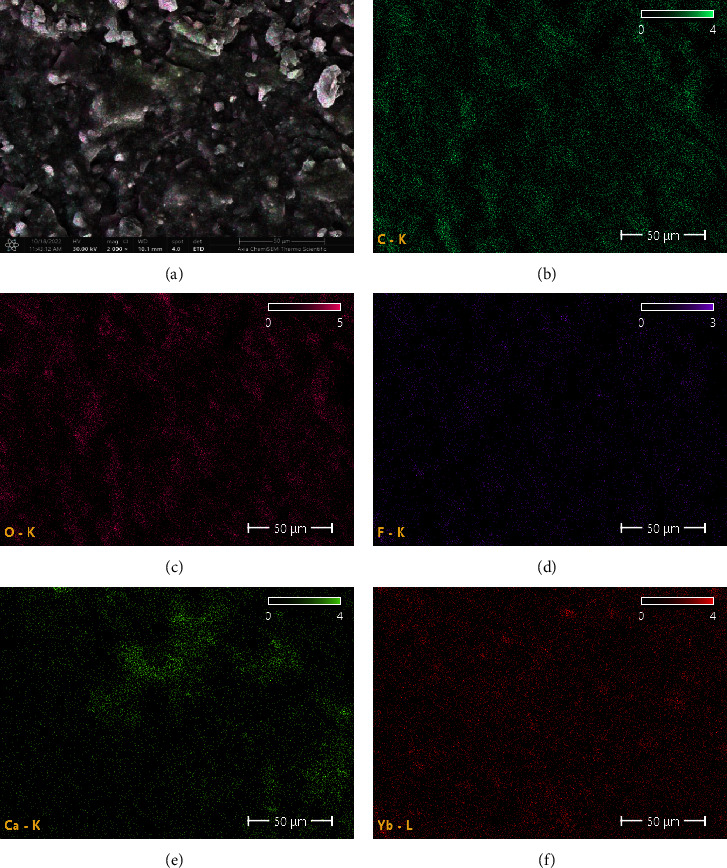
SEM-EDX micrographs of OnX 3D-printed material. (A) Dot mapping of an image illustrating the distribution of all elements in the sample. The interpretation of the color references of the image was contingent upon the colors in the subsequent photographs as follows: (B) dark green spots denote the presence of C element; (C) pink spots (O element); (D) purple spots (F element); (E) light green spots (Ca element); (F) red spots (Yb element; scale bar: 50 μm).

The EDX profile revealed distinct peaks related to Ytterbium (Yb), Carbon (C), Oxygen (O), Fluorine (F), and Calcium (Ca; Figure 6). While the peaks of C and O are related to the presence of an organic compound, the chief element peak of Yb element (19.4 wt%), which is an inorganic element, might indicate the potential nanofiller within the material. The SEM images, identified by the EDX, demonstrated the elemental details: one count map depicting the elements' relative compositions and capturing a granular rough surface (Figure 7A) and distribution maps of each element in the resin (Figure 7). The element density was indicated by its color brightness in mappings; the brighter the color, the more of the elements there are in the specimen. The mapping diagrams appeared as well-dispersed colored dots, indicating that the elements were evenly distributed throughout the specimen without particle aggregation. This could confirm the successful incorporation of nanoparticles into the polymer matrix. The EDX results were similar for the three specimens.

**Figure 8:**
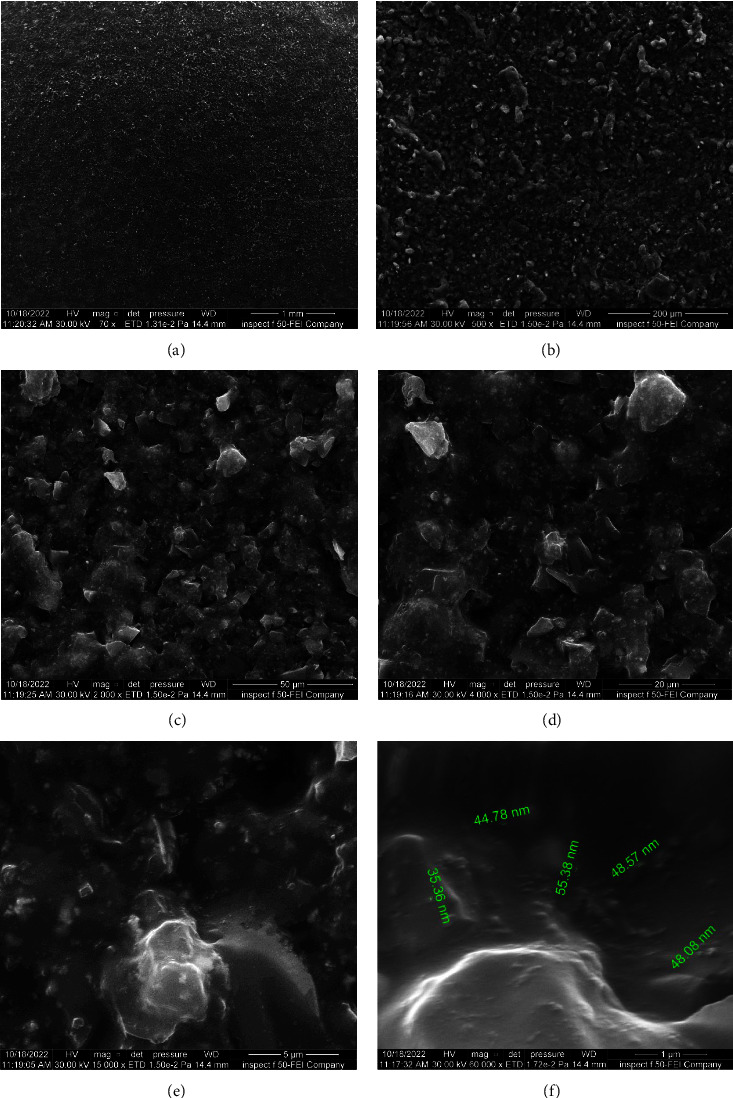
(A–F) FE-SEM micrographs of OnX 3D-printed material at different magnification powers.

At low magnifications (70x and 500x), FE-SEM photomicrographs revealed a consistent, homogenous, smooth surface with well-distributed small clumps of lighter gray scales. However, under higher magnifications (2000x, 4000x, and 15,000x), these features gradually transformed from a smooth to a somewhat rough surface, with an abundance of shallow dimples and irregular granules or clumps. Interestingly, at an even greater magnification (60,000x), a relatively smooth surface, except for inconspicuous irregularities with no defects, cracks, or precipitates, was shown. The nanoparticles' diameters ranged from 35.36 to 55.38 nm, with the majority possessing a smooth and spherical shape embedded within the matrix. Three specimens revealed similar FE-SEM patterns (Figure 8).

**Figure 9:**
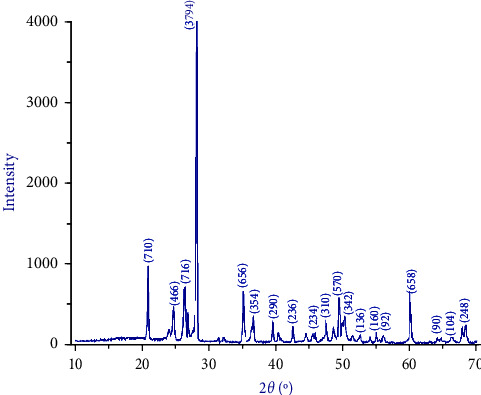
X-ray diffractogram of 3D-printed OnX material.

The XRD patterns for the three specimens were similar (Figure 9), with the diffraction signals ranging from 10° to 70°. Well-defined, sharp, and narrow diffraction peaks characterized OnX XRD patterns, all indicative of a crystalline material. The XRD characteristic diffraction peaks matched those of the standard reference pattern (JCPDS 65-3173) for Yb_2_O_3_, and these peaks were indexed accordingly. The major peak shown was at 2*Ɵ* = 26.65, and the principal crystallization peaks were at 2*Ɵ* = 20.95, 26.3, 35.1, 49.4, and 60. XRD findings also matched the FTIR and SEM-EDX results.

The data of the flexural properties were normally distributed. The average bending strength and the flexural modulus of OnX resin were 142.48 MPa and 7.842 GPa, respectively.

### 3.2. Mechanical Tests of the 3D-Printed Straight Wires

The one-way ANOVA and the Welch test demonstrated statistically significant differences among the groups for the load–deflection and pull-out tests.

#### 3.2.1. Load–Deflection Test

**Figure 10:**
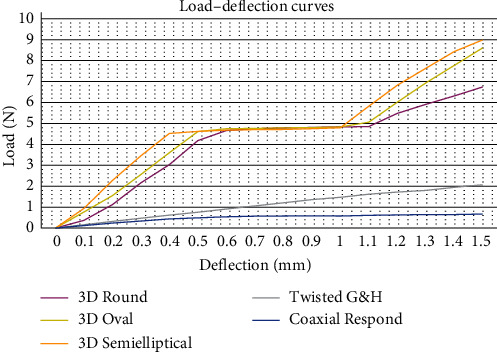
Load–deflection curves for retainer wire groups in a three-point bending test. Each line represents the mean value of 10 curves of each group obtained at intervals of 0.1 mm deflection.

At each tested deflection, the rank of the different wire groups in ascending order of load values was as follows: coaxial Respond, twisted G&H, 3D Round, 3D Oval, and last, 3D Semielliptical. All pairwise comparisons, at all deflection values, were statistically significant, except for deflections of 0.5 and 1 mm, between 3D Oval and 3D Semielliptical (Table 2). The 3D-printed wires displayed analogous load–deflection behavior over time. The 3D-printed wires initially showed that the load and deflection were proportional, but the curve subsequently flattened out and then steepened significantly (1–1.5 mm deflection). Three parameters were used to characterize the plateau: the main force exerted (about 4.5 N), the plateau length (about 0.6 mm), and the beginning and end of the plateau curve (about 0.4–0.5 mm deflection). On the contrary, twisted G&H and coaxial Respond revealed steadily increasing curves in which equivalent deflections could be achieved with considerably lower forces. (Table 2 and Figure 10).

#### 3.2.2. Pull-Out Test

**Figure 11:**
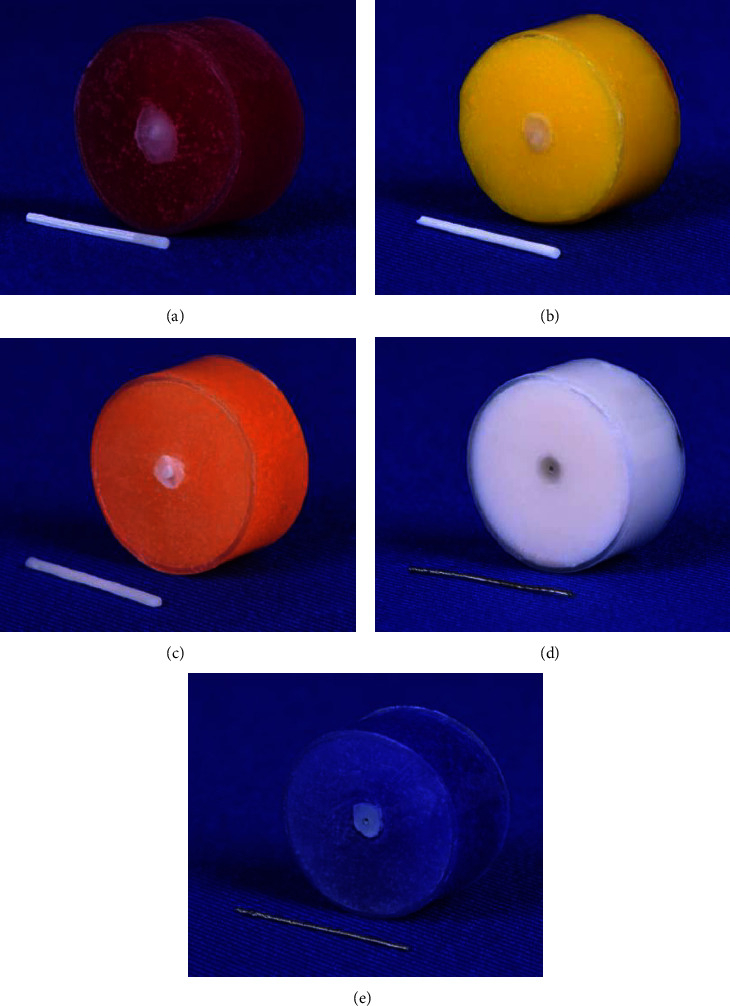
During the pull-out test, wires fractured in: (A) 3D Round; (B) 3D Oval; and (C) 3D Semielliptical, and detached from acrylic blocks in others: (D) Twisted G&H and (E) coaxial Respond.

Twisted G&H gave the greatest mean of the ultimate force of failure (N), parallel to 3D Semielliptical and 3D Oval, which did not exhibit any significant differences, while coaxial Respond revealed the lowest pull-out forces significantly. All 3D-printed wire groups showed wire fracture throughout the test, while the wires in the two MSW groups came out of the blocks (Figure 11). Among 3D-printed groups, 3D Oval and 3D Semielliptical demonstrated significantly greater values than 3D Round (Table 3).

## 4. Discussion

So far, consensus about the optimal fixed retainer is absent. Even though stainless steel MSW retainers remain the most common gold standard, they do possess notable drawbacks and restrictions [10, 32]. SprintRay OnX material was selected because, in October 2021, a new tooth shade nanoceramic hybrid 3D-printing resin was released and utilized to fabricate prosthetic denture teeth and provisional restorations. The manufacturer claims that SprintRay OnX has the best flexural strength and modulus in its category. This resin is biocompatible, FDA-approved, and has the perfect translucency and opacity to mimic natural dentition, making it exceptionally esthetic. Therefore, considering design and material, the study concept was to create a 3D-printed fixed retainer utilizing SprintRay OnX.

The average yield strength and modulus of elasticity in this study were consistent with that provided in the manufacturer's manual (147 MPa and 7.986 GPa). Firlej et al. [53] assessed the mechanical properties of NextDent C&B resin intended for use as a fixed lingual retainer. The flexural strength (70.8 MPa) and modulus of elasticity (1.95 GPa) were significantly lower. Comparable results had been reported in other research using commercially available 3D-printed dental resins (NextDent C&B resin [41, 54, 55] or A2 Everes Temporary [56]). This could affirm the manufacturer's alleged ability of the nanofiller of OnX material to enhance flexural properties.

In the load–deflection test, the 3D-printed wires demonstrated force levels many times higher than MSWs at all evaluated deflections. About twice as much force was needed concerning 1.5 mm deflection than 1 mm. The superior load–deflection rate might be desirable because it might highlight enough rigidity and dimensional stability to resist deformation from occlusal forces, minimizing the chance of relapse and inadvertent tooth movement (at deflection 1 and 1.5 mm). It is hypothesized that the lesser the force required to deflect the lingual retainer permanently, the easier the teeth can move [22]. In contrast, minor deformations (0.1 and 0.5 mm) of 3D-printed wires required, on average, forces of half or less of higher deflections (1 and 1.5 mm); this suggests that 3D-printed wires might have enough flexibility, although less than MSWs, to permit physiological tooth movement, thereby, promoting periodontal health and reduce the failure rate.

The 3D-printed wire groups displayed similar load–deflection behaviors over time. Initially, 3D-printed wires showed a proportionality between load and deflection (0.1–0.5 mm). Afterward, the curves flattened out, suggesting that relatively constant force values were required over an extended range of deformation. This nonlinear phenomenon could potentially be ascribed to the viscoelastic polymer behavior. The “plastic flow” is essentially an extension region of the typical stress–strain curve resulting from the rearrangement and reorientation of polymer chain segments, which establishes a new equilibrium state [57, 58]. The force exerted to reach the plateau and the plateau length were virtually the same in the three groups, that is, the curves nearly overlapping. Although there were minor differences in the start and end of the plateau across the three groups, it might cause little clinical concern. Since the plateau was beyond the typical limit of physiological tooth movement (0.5 mm), it has no clinical advantage.

The primary components of Transbond LR are silane-treated quartz, BIS-GMA, and TEGDEMA. Due to the similarity between the PMMA resin and the adhesive composite and the chemical nature of the adhesion between them, the bond strengths of the 3D-printed materials would be superior, as opposed to the MSWs' reliance on mechanical retention. However, in the present study, the mean ultimate forces of failure for 3D-printed wires fell between twisted G&H and coaxial Respond groups. However, the 3D-printed wire groups fractured cohesively without detachment from the adhesive. This could be attributed to the enhanced wire–composite bond strength and the wire resin's unfavorable strength being the weakest point. The chemical bond between the 3D-printed wires and the composite was apparently adequate to achieve satisfactory wire integration within the composite resin, thereby, fortifying the wire–composite complex. Interestingly, it might mitigate complications by facilitating the patient's detection so that he will seek treatment in a shorter time frame. On the contrary, adhesive-type fracture predominated for the twisted G&H and coaxial Respond, and the wires came out intact. This was owing to the absence of chemical bonding between the hydrophilic metal surface and the resin composite; the wire–composite interface seemed to be the central area of weakness. Although twisted G&H had a 0.0195″ cross-section (0.495 mm), much less than 3D-printed wires, it was seven-strand MSW as opposed to a single-strand 3D-printed wire and more mechanically interlocked than a smooth wire [59, 60], which might compensate for the greater cross-sectional area. On the other hand, Bearn et al. [50] found that increasing the thickness of the covering composite increased the force necessary to separate the wire from the composite. Nevertheless, there was negligible clinical benefit when the thickness was increased to more than 1.0 mm. Although the 3D-printed wires had a thinner composite, the bond between the wire and composite was more robust than that of the MSWs.

On the contrary, for coaxial Respond, the load–deflection curves were nearly flat, induced by far lighter forces, which were consistent with more pronounced deflections exhibited by previous studies [22, 51, 61], and provided the lowest detachment forces that were consistent with the findings of Baysal et al. [51] and Kadhum and Alhuwaizi [22] in a similar experimental setup. The greater the deformation before a failure, the greater the likelihood that the teeth may be repositioned without the composite breaking or wire rupturing and the possibility of composite crack propagation and bond failure [22, 62]. Baysal et al. [51] attributed the weak bond of Respond wires to a lack of micromechanical interlocking between the composite and the wire. This study reiterated that Respond wires might be unsafe for orthodontic retention.

Concerning the cross-sectional geometry, the load–deflection rates and the mean pull-out values for 3D Oval and 3D Semielliptical were significantly higher than 3D Round, probably due to the increased retainer's diameter and larger bonding surface area. As the cross-sectional area of the wire increases, so does the force needed to achieve a constant deflection [18, 45] and the ultimate force of failure during the pull-out test [22, 49, 50]. In broad terms, despite the differences among the three 3D-printed wire groups, the load–deflection changes were clinically trivial. The 3D Oval and 3D Semielliptical groups performed almost the same; nevertheless, 3D Semielliptical had slightly higher load–deflection slope values and pull-out forces. This might be attributed to the 3D Semielliptical wires' slightly increased bonding surface area for the pull-out test and different stress distribution patterns for the load–deflection test; however, there were clinically and statistically inconsequential differences. Given the more favorable load–deflection and bonding properties of 3D Oval and 3D Semielliptical, they were advocated over 3D Round.

FRC (fiber-reinforced composite) and milled PEEK retainers may be an effective esthetic alternative to traditional stainless steel MSW retainers [22, 63]. In the present study, the load–deflection values of 3D-printed groups were considerably lower than those of FRC retainers, with 0.75 and 1.2 mm diameters, utilizing 3PBT and a 14 mm span length [47]. However, the force levels of FRC retainers would be even higher if they had employed a span length of 10 mm, as was the case in this study. This indicates a greater degree of flexibility of 3D-printed wires than FRC, which permits the physiologic movement of teeth. On the other hand, the pull-out forces of 3D Semielliptical in this study (92.09 N) were much higher than those of milled PEEK retainers (0.8 mm) following air abrasion and conditioning with Visio.Link adhesive system (66.20 N) [22]. This result could be attributed to either the smaller cross-section of the PEEK retainer wires or the air abrasion scratches that weakened them, which was why they exhibited cohesive failure patterns.

## 5. Conclusions

The 3D-printed hybrid resin is a crystalline material that may consist of PMMA and uniformly distributed Yb elements. The flexural properties are in accordance with the manufacturer's measurements. Oval and semielliptical 3D-printed wires exhibited the most favorable load–deflection rate and ultimate forces of failure suggesting their potential superiority as lingual retainers. The result of this *in vitro* study should be interpreted cautiously, and further clinical research is warranted. Nevertheless, the preliminary results are particularly encouraging.

## Figures and Tables

**Table 1:** OnX 3D-printed material properties and specifications according to the manufacturer.

Specifications	SprintRay OnX properties by the manufacturer
Product identification	Photopolymer resin (nanoceramic hybrid class II 3D-printing)
Product class	A mixture of methacrylic acid esters, photoinitiators, proprietary pigment, and additive package (proprietary)
Flexural strength	147 MPa
Flexural modulus	7986 MPa
Impact strength	28 J/m
Miscibility with water	Nearly insoluble in water
Viscosity units, temp. (Brookfield)	220–250 cps at 25°C/77 F

**Table 2:** Descriptive and inferential statistics of load values (N) at different deflections.

Deflection	Groups	*n*	Mean	SD	Min	Max	Post hoc significance
0.1 mm	3D Round	10	0.374	0.010	0.36	0.39	All groups ^*∗*^
	3D Oval	10	0.774	0.014	0.75	0.79	All groups ^*∗*^
	3D Semielliptical	10	0.939	0.043	0.87	0.99	All groups ^*∗*^
	Twisted G&H	10	0.162	0.007	0.15	0.17	All groups ^*∗*^
	Coaxial Respond	10	0.120	0.006	0.11	0.13	All groups ^*∗*^

0.5 mm	3D Round	10	4.188	0.048	4.11	4.24	All groups ^*∗*^
	3D Oval	10	4.614	0.081	4.48	4.74	3D Round ^*∗*^ Twisted G&H^*∗*^ Coaxial Respond ^*∗*^
	3D Semielliptical	10	4.626	0.098	4.48	4.75	3D Round ^*∗*^ Twisted G&H^*∗*^ Coaxial Respond ^*∗*^
	Twisted G&H	10	0.766	0.010	0.75	0.78	All groups ^*∗*^
	Coaxial Respond	10	0.491	0.013	0.48	0.51	All groups ^*∗*^

1 mm	3D Round	10	4.380	0.030	4.33	4.42	All groups ^*∗*^
	3D Oval	10	4.783	0.038	4.73	4.83	3D Round ^*∗*^ Twisted G&H^*∗*^ Coaxial Respond ^*∗*^
	3D Semielliptical	10	4.798	0.046	4.73	4.86	3D Round ^*∗*^ Twisted G&H^*∗*^ Coaxial Respond ^*∗*^
	Twisted G&H	10	1.474	0.011	1.46	1.49	All groups ^*∗*^
	Coaxial Respond	10	0.589	0.017	0.56	0.62	All groups ^*∗*^

1.5 mm	3D Round	10	6.65	6.82	6.65	6.82	All groups ^*∗*^
	3D Oval	10	8.54	8.68	8.54	8.68	All groups ^*∗*^
	3D Semielliptical	10	8.68	9.20	8.68	9.20	All groups ^*∗*^
	Twisted G&H	10	2.04	2.10	2.04	2.10	All groups ^*∗*^
	Coaxial Respond	10	0.58	0.63	0.58	0.63	All groups ^*∗*^

^*∗*^The difference is significant at *p*  < 0.05.

**Table 3:** Descriptive and inferential statistics of the ultimate force of failure (N) in the pull-out test.

Groups	*n*	Mean	SD	Min	Max	Post hoc significance
3D Round	10	62.910	3.338	58.50	68.50	All groups ^*∗*^
3D Oval	10	88.240	3.717	83.80	95.00	Coaxial Respond ^*∗*^
3D Semielliptical	10	92.090	3.955	87.30	97.60	Coaxial Respond ^*∗*^
Twisted G&H	10	96.000	7.679	86.50	107.40	Coaxial Respond ^*∗*^
Coaxial Respond	10	48.090	1.081	46.50	49.70	All groups ^*∗*^

^*∗*^The difference is significant at *p*  < 0.05.

## Data Availability

The data used to support the findings of this study are available from the corresponding author upon request.
